# Apelin Promotes ECM Synthesis by Enhancing Autophagy Flux via TFEB in Human Degenerative NP Cells under Oxidative Stress

**DOI:** 10.1155/2020/4897170

**Published:** 2020-02-14

**Authors:** Wei Jiang, Pei Zhao, Xuemei Zhang

**Affiliations:** ^1^Department of Orthopedics, The First Affiliated Hospital of Chongqing Medical University, Chongqing 400016, China; ^2^Department of Obstetrics, The First Affiliated Hospital of Chongqing Medical University, Chongqing 400016, China

## Abstract

**Background:**

Apelin alleviates oxidative stress which contributes to the development of aging. IVDD is a disease closely correlated to aging and oxidative stress which is known to be harmful to NP cells' matrix synthesis. The purpose of the present study was to investigate the role and underlying mechanism of Apelin in NP cells' matrix degradation under oxidative stress.

**Methods:**

First, the mRNA and protein expressions of Apelin were checked by RT-PCR and Western blot in NP from normal and degenerative IVD to explore the relationship between Apelin and IVDD preliminarily. Then, H_2_O_2_ was used to mimic oxidative stress of NP cells. After treated with Apelin 13 and CQ, the GAG content was assessed by DMMB and the mRNA/protein expressions of NP matrix macromolecules (Collagen II and Aggrecan) and autophagy-related markers (LC3 and p62) were assessed by RT-PCR/Western blot. Finally, TFEB was knocked down by esiRNA-TFEB transfection and the nucleoprotein expression of TFEB and autophagy-related markers (LC3 and p62) were assessed by Western blot to discuss whether TFEB is involved in Apelin regulating autophagy flux in NP cells under oxidative stress.

**Results:**

Our data first confirmed that the mRNA and protein expressions of Apelin were decreased with IVDD. Furthermore, Apelin increased GAG content of NP cells and mRNA/protein expressions of NP matrix macromolecules (Collagen II and Aggrecan) and promoted autophagic flux (LC3II/I increased and p62 decreased) under oxidative stress. Finally, after transfected with esiRNA-TFEB, Apelin cannot promote autophagic flux any more in human degenerative NP cells.

**Conclusion:**

Our data indicated that Apelin promotes ECM synthesis by enhancing autophagy flux via TFEB in human degenerative NP cells under oxidative stress. This viewpoint may provide a new therapeutic idea for IVDD.

## 1. Introduction

Low back pain (LBP) has become a severe socioeconomic problem worldwide for its contribution to the drop in life quality and even disability in adults [1, 2]. Though the veritable cause of LBP is complex and unclear, many studies have demonstrated that intervertebral disc degeneration (IVDD) is a main cause of LBP [3–6]. Intervertebral disc (IVD) is consisting of nucleus pulposus (NP), fibrous annulus, and cartilage endplates. Changes in the NP were the earliest and most significantly during IVDD. Changes in the NP at the cellular and molecular levels will result in damage of structure and function of the extracellular matrix (ECM) and eventually lead to the loss of biomechanics and degeneration [7]. In light of this point, the maintenance of a healthy NP homeostasis may decrease the degradation of ECM and postpone the progression of the IVDD.

Apelin, a peptide that is 13 to 36 amino acids in length, has been confirmed to constitute a novel endogenous peptide system suggested to be involved in a broad range of physiological functions, including cardiovascular function, heart development, immunity, control of fluid homeostasis, glucose, and fat metabolism and obesity [8]. Recent studies indicated that Apelin is associated with aging. Apelin deficiency leads to multiple organs aging [9]. Considering that IVDD is closely related to aging, whether Apelin contributes to the process of IVDD is not clear.

Autophagy, a necessary cellular self-eating process, maintains the survival of cells under stress such as ischemia and hypoxia by scavenging senescent organelles and misfolded proteins [10, 11]. Substantial evidence including our early study has confirmed that autophagy is associated with an increase in the pathological processes of IVDD [12–14]. Activation of autophagy protected against apoptosis [14, 15] and enhanced ECM biosynthesis of NP cells [16]. Increasing evidence showed Apelin participates in autophagy process which contributes to antiaging [17–19] and alleviates oxidative stress which contributes to the development of aging [20–23] simultaneously. As the initiation and progression of IVD are closely associated with oxidative stress, the present study was to explore the relationship between Apelin and IVDD and further to assess whether Apelin protects oxidative damage-induced ECM decrease via autophagy activation.

## 2. Materials and Methods

### 2.1. Patients and NP Samples

**Figure 1:**
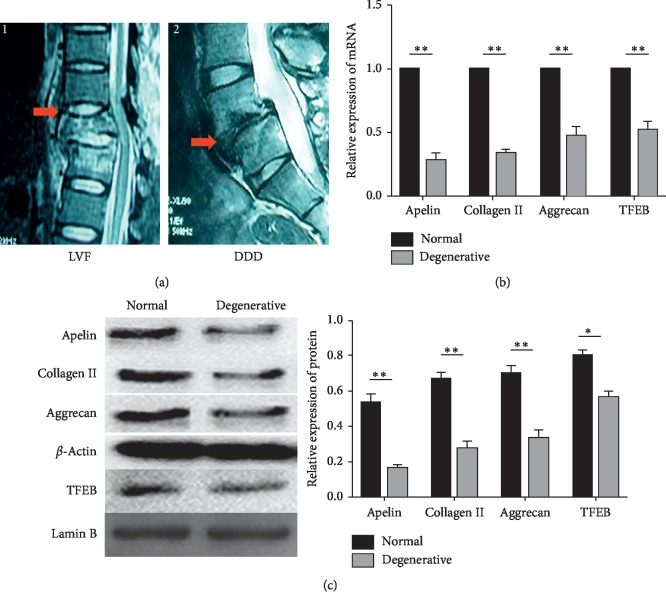
Low Apelin expression in NP of patients with DDD compared with that in NP of patients with LVF. (a) Representative MRI of patients. A1: patient with LVF; the red arrow indicates the experimental material position and the disc of T12/L1 is classified as grade I according to the Pfirrmann classification. A2: patient with DDD; the red arrow indicates the experimental material position and the disc of L5/S1 is herniated and classified as grade V according to the Pfirrmann classification. (b) RT-PCR analysis for Apelin, Collagen II, Aggrecan, and TFEB. The mRNA levels of Apelin, Collagen II, Aggrecan, and TFEB are decreased in NP cells from patients with DDD. (c) Western blot analysis for Apelin, Collagen II, Aggrecan, and TFEB (nucleoprotein) expression in NP cells from patients with LVF and DDD. The protein levels of Apelin, Collagen II, Aggrecan, and TFEB (nucleoprotein) are decreased in NP cells from patients with DDD. *n* = 3, ^*∗*^*p* < 0.05, ^*∗∗*^*p* < 0.01. All experiments were repeated at least three times. DDD: degenerative disc disease; NP: nucleus pulposus; LVF: lumbar vertebral fracture; TFEB: transcription factor EB.

The study complied with the Declaration of Helsinki and with approval from the Ethics Committee of Chongqing Medical University, and informed consent of all the patients involved in our study was obtained. The degeneration grade of IVD was classified according to the Pfirrmann classification [24] by magnetic resonance imaging (MRI) scan of spine prior to surgery. Normal NP tissues (grade I or grade II) were obtained from 4 patients with lumbar vertebral fracture (LVF) without formerly documented clinical history of LBP (3 women and 2 men; mean age, 32.30 ± 6.83 years) (Figure 1(a1)). The degenerative NP tissues (grades III-V) were obtained from 15 patients (9 women and 6 men; mean age, 35.26 ± 6.48 years) with degenerative disc disease (DDD) (Figure 1(a2)).

### 2.2. NP Cells Isolation and Culture

NP tissues and cells were isolated from human IVD samples as described in our previous study [14]. Next, the isolated NP cells were filtered and cultured into a monolayer in Dulbecco's modified Eagle's medium and Ham's F-12 medium (DMEM/F12, 1 : 1, Gibco, USA) supplemented with 15% FBS and 1% penicillin-streptomycin. To maintain the NP cells phenotype, the cells were cultured in a three-dimensional (3D) cell culture model by encapsulating them in Alvetex® (Reinnervate, Durham, UK) as reported [25] after the first passage. Briefly, the inserts were submerged in 70% ethanol for 10 min, washed twice with sterile water, incubated with poly-l-ornithine (1.5 *μ*g/ml) for 24 h, washed with phosphate buffered saline (PBS), and finally incubated in DMEM/F12 with 15% fetal bovine serum (FBS; Gibco, USA) and 1% penicillin-streptomycin for 2 h at 37°C and 5% CO_2_. The NP cells after first passage are seeded into 6-well plates containing Alvetex® inserts at a density of 1 × 10^6^ cells/mL.

### 2.3. Design of Experiment Groups

There were three main parts of experiments including NP tissues and cells in this study. For NP tissues experiments, two groups were designed: (i) normal NP tissues were obtained from patients' IVD with grade I or II according to the Pfirrmann classification by MRI and (ii) degenerative NP tissues were obtained from patients' IVD with grade V according to the Pfirrmann classification by MRI. All the NP cells for experiments were isolated from patients' IVD with grade III or IV according to the Pfirrmann classification by MRI. For NP cells experiments of stage one, three groups were designed: (i) control NP cells treated with H_2_O_2_, (ii) NP cells treated with H_2_O_2_ and Apelin 13, and (iii) NP cells treated with H_2_O_2_, Apelin 13, and chloroquine (CQ). For NP cells experiments of stage two, four groups were designed: (i) control NP cells treated with H_2_O_2_, (ii) NP cells treated with H_2_O_2_ and Apelin 13, (iii) NP cells treated with H_2_O_2_ and small interfering RNA- (esiRNA-) transcription factor EB (TFEB), and (iiii) NP cells treated with H_2_O_2_, esiRNA-TFEB and Apelin 13. All NP cells were treated with H_2_O_2_ (100 *μ*g/mL) as the oxidative damage. Apelin 13 (100 nM) was applied to investigate its protective effects. CQ (40 *μ*M) was added along with the culture medium to investigate the role of autophagy in this process. The esiRNA-TFEB was applied to investigate the possible mechanism of Apelin regulating autophagy in NP cells under oxidative stress.

### 2.4. RNA Extraction and Real-Time RT-PCR

Total RNA was extracted from human NP tissues and cells using TRIZOL@ Reagent (Invitrogen, 15596-018) according to the manufacturer's instruction. An ultraviolet spectrophotometer (Olympus, Japan) was used to measure the purity and concentration of RNA. Next, the isolated RNA samples were used to synthesized cDNA using PrimeScript@ RT reagent Kit With gDNA Eraser (Takara, RR047Q). Then, real-time PCR (3 min at 95°C, followed by 40 amplification cycles of 15 s at 95°C, respective annealing temperature for 20 s, and an elongation phase 20 s at 72°C) was performed on a real-time PCR machine (Applied Biosystems, USA) with the method of SYBR Green detection chemistry (TOYOBO, QPK-201). The primers were synthesized by TaKaRa (TaKaRa, China) and shown in Table 1. The fold-change in gene expression relative to the control was calculated by 2^∆∆CT^.

### 2.5. Western Blot Analysis

Nuclear and Cytoplasmic Protein Extraction Kit (ComWin Biotechnology, P0028) was used to extract the protein of NP tissues and cells according to the manufacturer's instruction. NP cells were washed in ice-cold PBS and lysed using RIPA Lysis Buffer (Beyotime, P0013B). The lysates were separated on an sodium dodecyl sulfate/polyacrylamide gel electrophoresis (SDS/PAGE) and transferred onto the polyvinylidene difluoride (PVDF) membrane (Beyotime, China). The membranes were blocked with 5% nonfat dry milk in tris-buffered saline (TBST) for 1 h and incubated with primary antibodies: rabbit anti-Apelin (1 : 1000), rabbit anti-Collagen II (1 : 1000), mouse anti-Aggrecan (1 : 1000), rabbit anti-TFEB (1 : 1000), rabbit anti-LC3 (1 : 1000), rabbit anti-p62 (1 : 1000), mouse anti-Lamin B (1 : 500), and mouse anti-*β*-actin (1 : 500) overnight at 4°C. After washed three times for 10 min in TBST, the membranes were incubated in secondary antibody, goat anti-rabbit IgG-HRP (1 : 5000) and goat anti-mouse IgG-HRP (1 : 5000) for 1 h. Finally, the membranes were treated with ECL plus reagent (Invitrogen, WP20005) and the results were analyzed by the software.

### 2.6. Transfection

In view of the low transfection efficiency of cells after 3D culture in our preliminary test, the endoribonuclease prepared small interfering RNA- (esiRNA-) TFEB was transfected into NP cells in a monolayer culture before being seeded into Alvetex® inserts. The esiRNA for human TFEB gene silencing was purchased from Sigma-Aldrich (EHU059261). For transfection, cells were seeded in 6-well plate for 24 h and transfected with esiRNA-TFEB using PepMute siRNA Transfection Reagent (SignaGen, USA) according to the manufacturer's instructions. After 72 hours of transfection, NP cells were seeded into 6-well plates containing Alvetex® inserts.

### 2.7. Glycosaminoglycan Content Measurement

The 1,9-dimethylmethyleneblue (DMMB) assay was applied to measure the glycosaminoglycan (GAG) content of the cultured NP cells as described in a previous report [26]. Briefly, the NP cells were collected and digested by 5 mg/ml papain (Sangon, Biotech Co., Ltd., China) for 6 h at 60°C. Then, the GAG content in the digested solution was calculated according to the absorbance value at 525 nm. Additionally, the shark cartilage chondroitin sulfate (Sigma, USA) was used as a standard in the DMMB assay.

### 2.8. Statistical Analysis

Data were expressed as mean ± standard deviation for three independent experiments. Statistical differences were measured with Student's *t*-test for comparison between two groups or an analysis of variance (ANOVA) followed by Turkey's *t*-test for comparison of multiple groups. SPSS 14.0 statistical software program (SPSS Inc., IL, USA) was used for statistical analyses, and *p* < 0.05 was considered statistically significant.

## 3. Results

### 3.1. Low Apelin Expression in NP of Patients with DDD Compared with That in NP of Patients with LVF

In order to confirm the relationship between Apelin and IVDD, RT-PCR/Western blot was applied to measure the mRNA/protein expression of Apelin of NP from LVF and DDD. Our data showed that both mRNA and protein expressions of Apelin decreased obviously in NP from DDD compared to those in NP from LVF. As the most essential transcription factor of autophagy, the mRNA expression of TFEB and the nucleoprotein expression of TFEB in NP from LVF and DDD were measured. The data showed that they were also decreased obviously in NP from DDD compared to those in NP from LVF. We further detected the expressions of Collagen II and Aggrecan with the same methods; the results showed that both expressions of Collagen II and Aggrecan were reduced significantly in NP from DDD (Figures 1(b) and 1(c)).

### 3.2. Apelin Promotes ECM Synthesis of Degenerative NP Cells under Oxidative Stress

**Figure 2:**
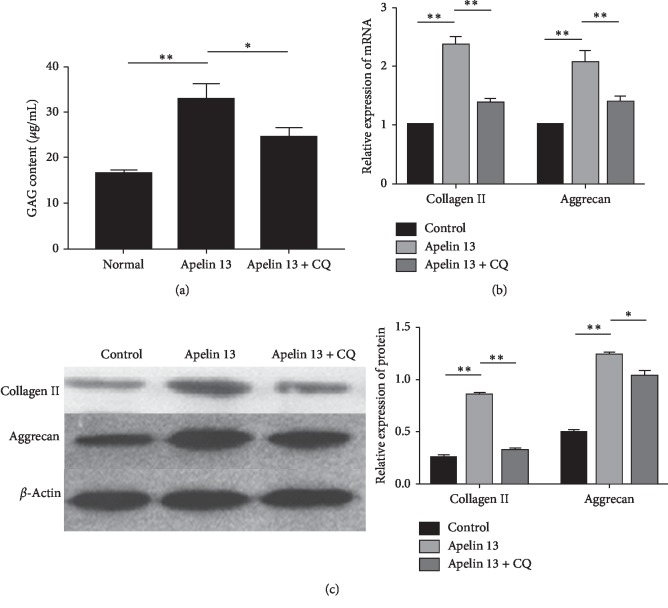
Apelin promotes ECM synthesis of degenerative NP cells under oxidative stress. (a) DMMB analysis for GAG content after treatment of Apelin 13/CQ under oxidative stress. The GAG content is increased after Apelin 13 treatment, and they are partly decreased after autophagy is inhibited by CQ. (b) RT-PCR analysis for Collagen II and Aggrecan after treatment of Apelin 13 or/and CQ under oxidative stress. The mRNA levels of Collagen II and Aggrecan are increased after Apelin 13 treatment, and they are partly decreased after autophagy is inhibited by CQ. (c) Western blot analysis for Collagen II and Aggrecan after treatment of Apelin 13/CQ under oxidative stress. The protein levels of Collagen II and Aggrecan are increased after Apelin 13 treatment, and they are partly decreased after autophagy is inhibited by CQ. ^*∗*^*p* < 0.05, ^*∗∗*^*p* < 0.01. All experiments were repeated at least three times. ECM: extracellular matrix; NP: nucleus pulposus; DMMB: 1,9-dimethylmethyleneblue; GAG: glycosaminoglycan; CQ: chloroquine.

In order to explore the role of Apelin for ECM synthesis of degenerative NP cells under oxidative stress, DMMB assay was applied to measure the GAG content of the NP cells and RT-PCR/Western blot were used to assess the mRNA/protein expressions of NP matrix macromolecules (Collagen II and Aggrecan). The results showed that the GAG content was increased obviously and the expressions of Collagen II and Aggrecan were significantly upregulated at both mRNA and protein levels in the Apelin 13 group compared with the control group. After autophagy was inhibited by CQ, the expression levels of GAG, Collagen II, and Aggrecan were partly decreased (Figures 2(a)–2(c)).

### 3.3. Apelin Enhances Autophagy in Degenerative NP Cells under Oxidative Stress

**Figure 3:**
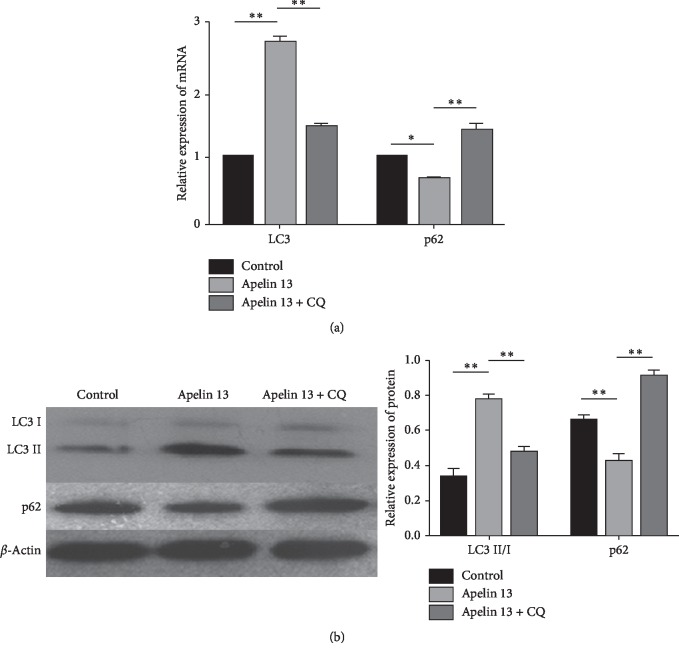
Apelin enhances autophagy flux in degenerative NP cells under oxidative stress. (a) RT-PCR analysis for LC3 and p62 after treatment of Apelin 13 /CQ under oxidative stress. The mRNA levels of LC3 are increased and p62 is decreased after Apelin 13 treatment; and LC3 is partly decreased and p62 is partly increased After autophagy is inhibited by CQ. (b) Western blot analysis for LC3II/I and p62. The protein level of LC3II/I is increased and p62 is decreased after Apelin 13 treatment; and LC3II/I is partly decreased and p62 is partly increased after autophagy is inhibited by CQ. ^*∗*^*p* < 0.05, ^*∗∗*^*p* < 0.01. All experiments were repeated at least three times. NP: nucleus pulposus; CQ: chloroquine.

In order to explore the role of Apelin for autophagy in degenerative NP cells under oxidative stress, RT-PCR/Western blot were used to assess the mRNA/protein expressions of cellular autophagy-related markers (LC3 and p62). The results showed that the mRNA of LC3 and the protein of LC3II/I were significantly upregulated in the Apelin 13 group compared to those in the control group. However, the autophagy inhibitor CQ partly decreased their expression when it was added along with the culture medium in the Apelin 13-treated degenerative NP cells. The mRNA and protein expressions of p62 were significantly downregulated in the Apelin 13 group compared with those in the control group. However, Apelin 13 failed to reverse increased p62 caused by CQ (Figures 3(a) and 3(b)).

### 3.4. Apelin Promotes Autophagy via TFEB in Degenerative NP Cells under Oxidative Stress

**Figure 4:**
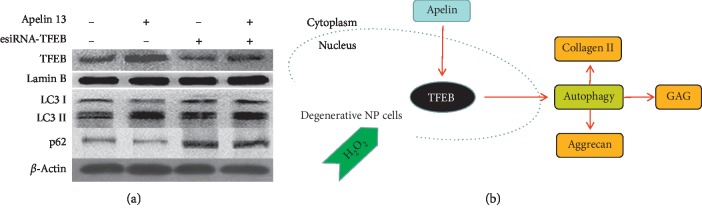
Apelin promotes autophagy via TFEB in degenerative NP cells under oxidative stress. (a) Western blot analysis for TFEB (nucleoprotein), LC3II/I, and p62 after autophagy was inhibited by esiRNA-TFEB. The protein levels of TFEB (nucleoprotein) and LC3II/I are decreased and p62 is increased after treated with esiRNA-TFEB. Apelin 13 fails to rescue autophagy flux inhibition caused by esiRNA-TFEB. (b) Schematic representation of proposed mechanism of Apelin promotes ECM synthesis of human degenerative NP cells under oxidative stress by activating autophagy flux via TFEB. All experiments were repeated at least three times. TFEB: transcription factor EB; NP: nucleus pulposus; ECM: extracellular matrix.

The esiRNA-TFEB was used to confirm whether Apelin promotes autophagy via TFEB in degenerative NP cells. Western blot results showed that esiRNA-TFEB could inhibit nucleoprotein expression of TFEB and autophagy (LC3II/I decreased and p62 increased). However, Apelin failed to reverse the effects of autophagy inhibition caused by esiRNA-TFEB (Figure 4(a)).

## 4. Discussion

Substantial evidence indicated that IVDD is a main contributor to LBP. Although the mechanisms involved in the pathogenesis of IVDD have not yet been clearly understood, NP matrix degradation or dyssynthesis is one of the most common features during IVDD. An adequate NP matrix production is implicated to maintain the disc's mechanical property and thus the spinal stability, and decreased NP ECM leads to deteriorated IVDD [27]. In this study, we detected Collagen II and Aggrecan, the two macromolecules within the NP matrix in human NP from LVF and DDD. Results confirmed that both gene and protein expressions of Collagen II and Aggrecan were decreased obviously in NP from DDD than those in LVF. Oxidative stress is known to be closely correlated with disc pathogenesis and progression; moreover, it is harmful to disc cell's normal biology, including matrix synthesis and cell viability [28]. Therefore, H_2_O_2_ was used to mimic oxidative but not cytotoxic environment in the present study.

Apelin, an endogenous ligand for the G protein-coupled receptor APJ, is widely expressed in various organs [8]. Recent research has indicated that Apelin is downregulated with age and that its absence accelerates the onset and progression of aging [9, 29–31]. Moreover, Apelin alleviates oxidative stress which contributes to the development of aging. Consider that (1) IVDD is a disease that is closely related to aging, (2) oxidative stress is known to be closely correlated with disc pathogenesis and progression, and (3) oxidative stress is harmful to disc cell's matrix synthesis. Hence, we explored the relationship between Apelin and IVDD and further to confirm whether Apelin regulates degenerative NP cell's matrix synthesis under oxidative stress. We first found that the gene and protein levels were obviously decreased in NP from degenerative IVD compared to those from normal IVD. Apelin could promote gene and protein expressions of Collagen II and Aggrecan under oxidative stress. This is the first report of Apelin roles in human NP cells. It is a little bit of contradiction to Hu's [32] report about the catabolic role of Apelin on articular cartilage. We considered the different cell types (human NP cells versus rat articular chondrocytes) and cell status (degenerative versus normal) as the main reasons of contrary results.

Many researches have indicated that autophagy plays important roles in IVDD and our early study also confirmed that autophagy activation could inhibit apoptosis of degenerative human NP cells [12–15]. Recent studies indicated that Apelin participates in autophagy process which contributes to antiaging: Apelin 13 induces autophagy and reduces the accumulation of lipid in foam cells [17], Apelin 13 significantly increases expression of LC3-II/I and beclin-1 in H9c2 cardiomyocytes [18], and Apelin increases the viability of arterial disease-mesenchymal stem cells by promoting protective autophagy [19]. In the present study, we found that Apelin 13 increases expression of LC3II/I and decreases expression of p62; after autophagy inhibition by CQ, the effects of Apelin 13 promoting ECM synthesis (including Collagen II, Aggrecan, and GAG) were weakened. These results indicated that Apelin 13 promotes ECM synthesis of human degenerative NP cells under oxidative stress through activating autophagy flux.

Transcription factor EB (TFEB), a member of microphthalmia-associated transcription factor/transcriptional factor E family, has been identified as a master regulator of autophagy flux via inducing lysosome biogenesis and promoting autophagosome formation as well as its fusion with lysosome [33–35]. Recent research indicated that TFEB protects nucleus pulposus cells against apoptosis and senescence via restoring autophagic flux [36]. However, whether Apelin regulates human NP cells autophagy via TFEB has not been reported. In the present study, after autophagy flux was inhibited by TFEB downregulation via esiRNA-TFEB, Apelin 13 failed to increase the expression of LC3II/I and reduce the expression of p62. These results confirmed that Apelin enhances autophagy flux via TFEB activation in human degenerative NP cells.

In conclusion, the present study confirmed that Apelin was decreased with IVDD, and Apelin could promote ECM synthesis of NP cells under oxidative stress by activating autophagy flux via TFEB (Figure 4(b)). This viewpoint may provide a new therapeutic idea for IVDD.

## 5. Conclusions

Apelin promotes ECM synthesis of human degenerative NP cells under oxidative stress by activating autophagy flux via TFEB.

## Figures and Tables

**Table 1:** Primer sequence for target gene.

Gene	Primer sequence
*β*-Actin	Forward: 5′-TCACCATGGATGATGATATCGC-3′
	Reverse: 5′-CGTGCTCGATGGGGTACTTCA-3
Apelin	Forward: 5′-CTGCTCTGGCTCTCCTTGAC-3′
	Reverse: 5′-ATGGGTCCCTTATGGGAGAG-3′
Collagen II	Forward: 5′-TTCAGCTATGGAGATGACAATC-3′
	Reverse: 5′-AGAGTCCTAGAGTGACTGAG-3′
Aggrecan	Forward: 5′-ATGCCCAAGACTACCAGTGG-3′
	Reverse: 5′-TCCTGGAAGCTCTTCTCAGT-3′
TFEB	Forward: 5′-GGAGGCGTGATGGTGAACTC-3
	Reverse: 5′-CTCACAACAGCCTGTGAGGT-3′
LC3	Forward: 5′-GTGAGTGTGTCCACGCCCAT-3′
	Reverse: 5′-AGGTTTCCTGGGAGGCGTAG-3′
P62	Forward: 5′-TGCCCCTCTTCTGTCTCATAGT-3′
	Reverse: 5′-CACTTGTTTTGCTGCCCTAAAT-3′

## Data Availability

All the data used to support the findings of this study are included within the article.
